# Laparoscopic metabolic surgery for the treatment of type 2 diabetes in Asia: a scoping review and evidence-based analysis

**DOI:** 10.1186/s12893-018-0406-3

**Published:** 2018-09-17

**Authors:** Zhiyong Dong, Sheikh Mohammed Shariful Islam, Ashley M. Yu, Rui Qu, Bingsheng Guan, Junchang Zhang, Zhao Hong, Cunchuang Wang

**Affiliations:** 1 Department of Bariatric Surgery, the First Affiliated Hospital of Jinan University, No.613, Huangpu Avenue West, Guangzhou, 510630 China; 2 Institute for Physical Activity and Nutrition (IPAN), Deakin University, Melbourne, VIC 3125 Australia; 3 Faculty of Medicine, University of Ottawa, Ottawa, Canada; 4 Robert H Lurie Medical Research Center, Feinberg School of Medicine, Northwestern University, Chicago, IL 60611 USA

**Keywords:** Metabolic surgery, Type 2 diabetes, Obesity, BMI < 35 kg/m^2^, evidence-based analysis

## Abstract

**Background:**

Laparoscopic metabolic surgery has been previously shown to be an effective treatment for obese patients with type 2 diabetes (T2DM). The objective of this scoping review is to determine the impact of metabolic surgery for the treatment of type 2 diabetes in Asia and perform an evidence-based analysis.

**Methods:**

We performed a literature search in PubMed for research on laparoscopic metabolic surgery for the treatment of T2DM in Asia region. We classified the included studies based on the *Oxford Center for Evidence Based Medicine* guidelines. And performed and evidence analysis.

**Results:**

In total, 205 articles were identified. 62.9% of the studies were from East Asia. The evidence of 26 studies are level I, 59 are level II. Laparoscopic sleeve gastrectomy (LSG) was the most commonly reported surgical procedure (63.1%) in Asia. The number of laparoscopic metabolic surgery for T2DM in Asian countries has increased rapidly over the last 8 years. We identified 16 studies which showed that laparoscopic metabolic surgery is an effective and safe treatment for T2DM in patients with a BMI of > 25 kg/m^2^ to < 35 kg/m^2^ in Asia.

**Conclusions:**

Our results suggest that laparoscopic metabolic surgery might be an effective and safe treatment for T2DM patients with BMI < 35 kg/m^2^, and that LSG is the most commonly performed surgical procedure for this in Asia.

## Background

The prevalence of type 2 diabetes mellitus (T2DM) and obesity is increasing rapidly worldwide, especially in developing countries [1]. The International Diabetes Federation reportedin 2015 that there were 450 million diabetic patients globally [2]. 85–95% of the patients had T2DM, and 75% were from low-income countries [2]. Ng et al. [3] evaluated that the total number of obese persons were 671 million all over the world. 13% of them resided in the United States and 15% in China and India. Obesity is recognized as one of the major risk factor for diabetes, coronary heart diseases, stroke, sleep apnea and cancer [4–6]. It is well known that uncontrolled obesity can lead to significant health problems, decrease life expectancy, and influence quality of life. Obesity and co-morbid diabetes imposes a significant global burden to health systems, societies, families, and individuals affected [7, 8].

Medical therapy can be of limited value in the treatment of obese patients with T2DM [9–11]. Wittgrove et al. [12] performed the first five cases of laparoscopic gastric bypass surgery in 1993–1994 which showed promising results of weight loss. Since then, laparoscopy has become the main method of bariatric surgery. In 1995, Walter Porieset al. [13] found that bariatric surgery can significantly reduce weight. In this same study, 83% of T2DM patients, and 98% of patients with impaired glucose tolerance, experienced postoperative normalization of blood sugar, serum insulin, and glycosylated hemoglobin, following surgery, and a long-term stabilization of T2DM remission [13]. Evidence shows that bariatric surgery not only control a patient’s weight, but can also improve obesity-related complications, including hypertension, hyperlipidemia, snoring, sleep apnea syndrome, polycystic ovarian syndrome, and other metabolic diseases, especially T2DM [14–18]. At present, the common metabolic surgical procedures performed are: laparoscopic sleeve gastrectomy (LSG), laparoscopic adjustable gastric banding surgery (LAGB), laparoscopic biliopancreatic diversion with duodenal switch (BPD-DS), and laparoscopic Roux-en-Y gastric bypass (LRYGB) [19–21]. The most effective metabolic surgical procedures are the RYGB and duodenal switch [19–21].

Several reports of laparoscopic metabolic surgery for T2DM are available in different Asian countries. However, information regarding which surgical methods are most suitable for T2DM in Asia is scarce. Therefore, we performed this scoping review and evidence-based analysis to understand the current and common bariatric surgical procedures for T2DM in Asia.

## Methods

### Search strategy

We conducted a literature search from the electronic databases of PubMed from 1st January, 1994 to 19st November, 2017. The following key search terms were used: “bariatric surgery OR metabolic surgery OR obesity surgery OR weight loss surgery” AND “diabetes mellitus” AND “names of Asian countries and regions: according to WHO list of countries and regions.” There were no language restrictions.

### Screening

Three authors were involved in the independent title and abstract screening of potentially relevant articles. All screening was performed independently and in duplicate. If there were any discrepancies between authors and a consensus was unable to be reached, a fourth author was consulted.

### Inclusion and exclusion criteria

Studies describing laparoscopic metabolic surgery for T2DM, in patients with or without obesity were included. Studies were included if they were conducted in one of 49 Asian countries. We excluded all studies from non-Asian countries, patients without type 2 diabetes, and any non-laparoscopic surgeries.

### Literature screening and data extraction

Three reviewers (DZ, QR, GB) independently extracted the following terms from each study: year of publication, the country of first author, regions (East Asia, Southeast Asia, South Asia, West Asia, Central Asia), title, presence of patients with or without T2DM, language, type of bariatric surgery, and the type of clinical research (animal trials, basic research). All data was extracted in duplicate, and any disagreements of data extractionwere resolved through discussion between authors.

### Evidence grade criteria and statistical analysis

Three reviewers (DZ, QR, GB) independently classified the clinical study, using *Oxford Center for Evidence Based Medicine (EBM) Levels of Evidence* guidelines (https://www.cebm.net/2017/05/ocebm-levels-of-evidence/) [22]. Any difference between the reviewers was resolved by discussion. Data extraction was performed using Microsoft Excel 2007 software.

## Results

### Literature search

**Fig. 1:**
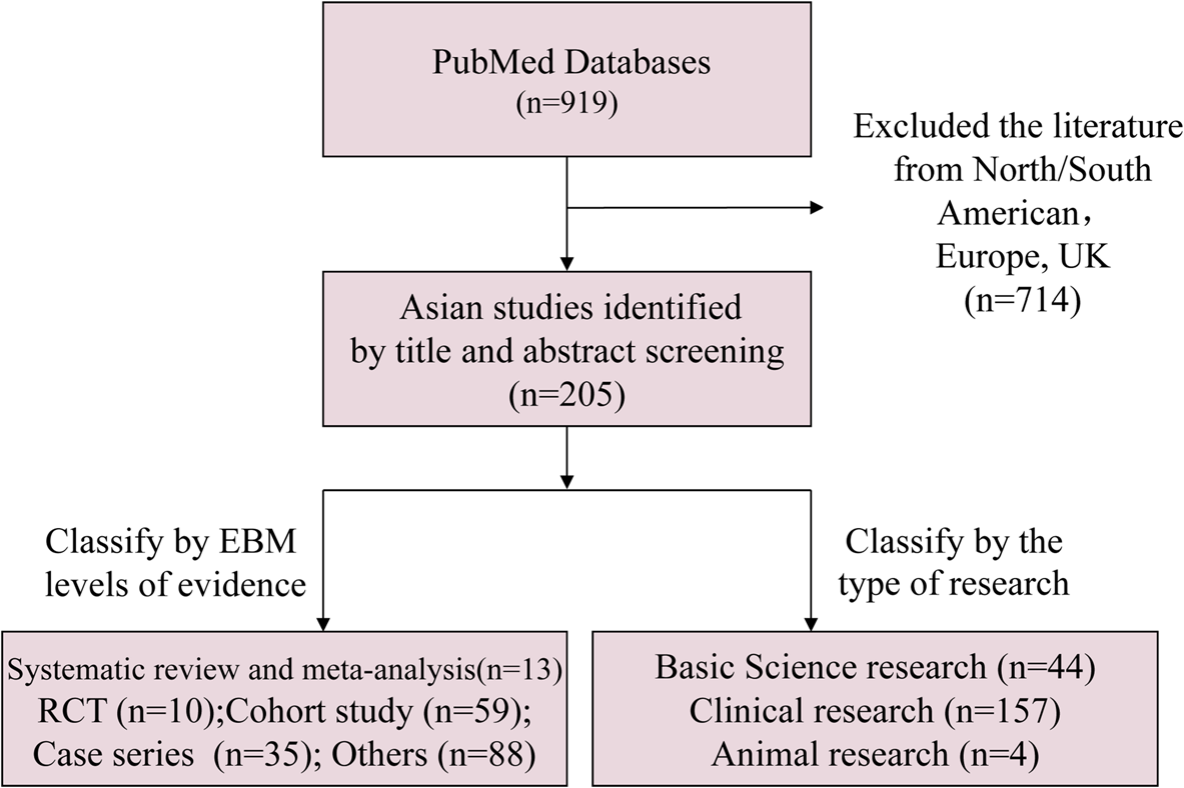
Flow diagram of the search process and study classification

Our search yielded 919 articles (including 102 systematic review/meta-analyses, 52 randomized controlled trials (RCTs), 398 cohort studies, 129 case series, 238 case reports and others) from all over the world, and 205 articles in Asian countries (including 13 systematic review/meta-analyses, 10 RCTs, 59 cohort studies, 35 case series, and 88 case report and others). Of the 205 reports included, 157 were clinical research, 44 were basic research, and four animal trials. Our study flow diagram is shown in Fig. 1.

### Laparoscopic metabolic surgery for T2DM in Asia

**Fig. 2:**
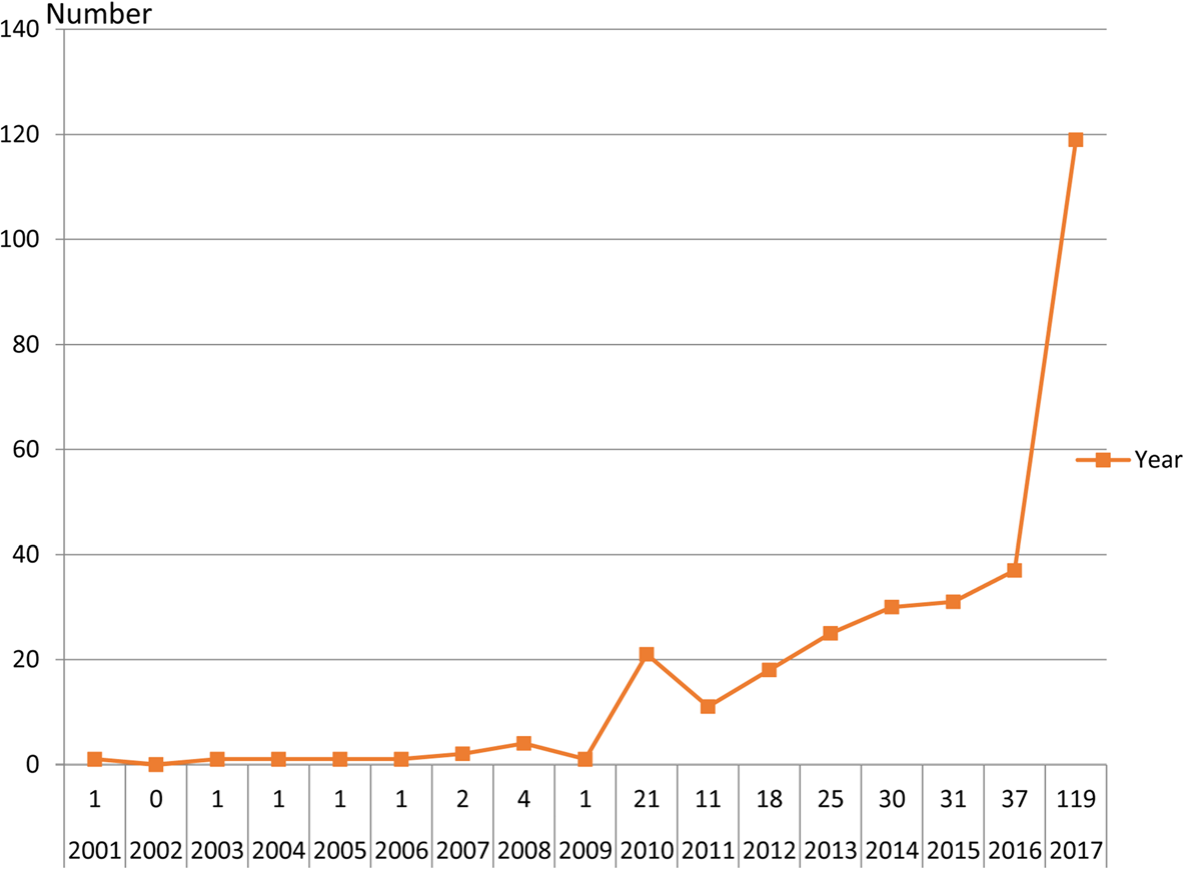
The trends and numbers of literature on laparoscopic metabolic surgery for T2DM in Asia

From our search, there is a trend of increased reporting of literature on laparoscopic metabolic surgery for T2DM in Asian countries and regions over the past decade, as shown in Fig. 2.

### The distribution of reports on laparoscopic metabolic surgery for T2DM in Asia

**Fig. 3:**
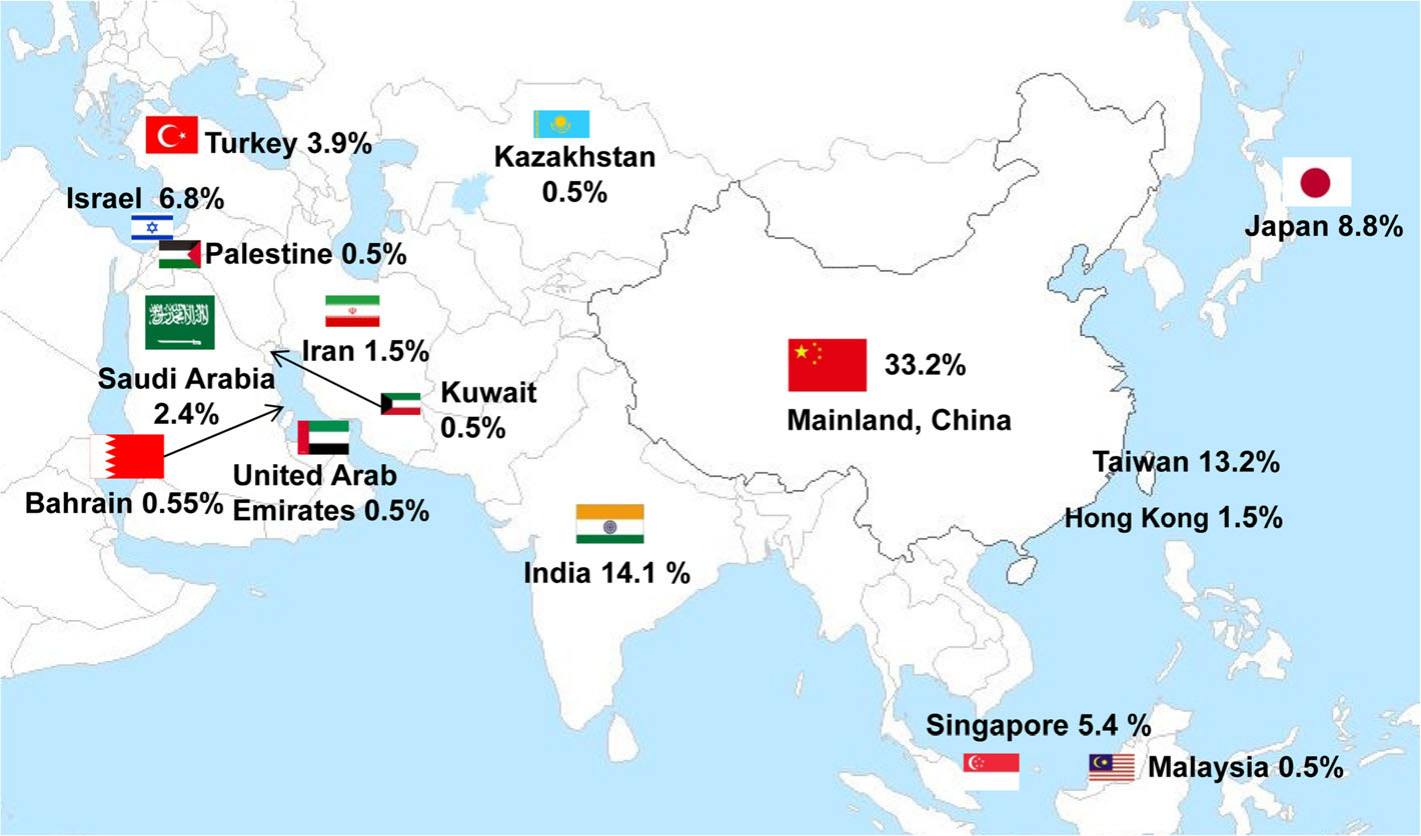
The distribution and rate of literature on laparoscopic metabolic surgery for T2DM in country of Asia. (The initial map courtesy of "map.ps123.net")

The distribution and proportion studies were: 129 papers from East Asia (62.9%), mostly from China, Japan, and South Korea; 12 papers from Southeast Asia (5.9%), located in Singapore, and Malaysia; 29 papers from South Asia (14.1%), distributed in India; and 34 papers from West Asia (16.5%), representing Israel, Turkey, and Saudi Arabia. Only one paper represented Central Asia (0.5%) from Kazakhstan. These results are presented in Fig. 3.

### The level of clinical study on laparoscopic metabolic surgery for T2DM in Asia

Clinical studies were classified according to the Oxford Center for EBM Levels of Evidence guidelines: Level I 1a (systematic review/ meta-analysis) - 13 articles were published in Asia out of 102 globally, accounting for 12.7%; Level I 1b (RCT) - 10 out of 52 globally (19.2%); Level II 2b (cohort study) - 59 out of 398 globally (14.8%); Level IV 4 (case series) - 35 out of 129 globally (27.1%). Of the 238 case reports or other articles published globally representing Level V 5 evidence, 88 were from Asia (37.0%) (Table 1).

**Table 1:** The level of included clinical study on laparoscopic metabolic surgery for T2DM

Levels	Type of study	No. of World	No. of Asian	Rates
I 1a	Systematic review/ meta-analysis	102	13	12.7% (13/102)
I 1b	RCT	52	10	19.2% (10/52)
II 2b	Cohort study	398	59	14.8% (59/398)
IV 4	Case series	129	35	27.1% (35/129)
V 5	Case report/other	238	88	37.0% (88/238)

### The procedures of clinical study on laparoscopic metabolic surgery for T2DM in Asia

A total of 13,742 patients were reported to have undergone laparoscopic metabolic surgery for T2DM from the included studies in Asia. 3752 (27.3%) cases were performed by LRYGB, and majority in East Asia (53.2%). 8242 (60.0%) cases by LSG, 70.4% in East Asia. 653 (4.8%) by LGAB, and 1095 (8.0%) by other metabolic surgery procedures.

### Clinical study of laparoscopic metabolic surgery for T2DM with BMI of > 25 kg/m^2^ to < 35 kg/m^2^ in Asia

There were two RCTs, one cohort study, and 14 case series whereby a total of 507 cases were performed by LRYGB, 172 by MGB, 107 by LSAGB, 57 by LJISSA and 51 by LSG. 16 studies were on laparoscopic metabolic surgery for T2DM with BMI of > 25 kg/m^2^ to < 35 kg/m^2^ in Asia (Table 2) [22–37]. Thirteen studies were from China, two from India, and one from South Korea. All the 16 studies reported a different rate, ranging from 53 to 96.2% of remission of diabetes in patients with T2DM and BMI of > 25 kg/m^2^ to < 35 kg/m^2^. There were no reported mortalities in any of the trials, post-operative follow-up times ranging from 1 week up to 5 years (Table 3). Moreover, the major complications were incomplete intestinal obstruction, mild upper gastrointestinal bleeding, excessive postoperative suture line bleeding with shock, anastomotic ulceration, anemia, low albumin, bile reflux, excess weight loss, early hemorrhage, marginal ulcers, seromas, nausea, vomitingand diarrhea, gastric fistula and infection, all complications were cured by symptomatic treatment (Table 3). These results suggest that laparoscopic metabolic surgery may be effectively used to treat T2DM in patients with BMI of > 25 kg/m^2^ to < 35 kg/m^2^ in Asia [23–38].

**Table 2:** The characteristic of clinical study on laparoscopic metabolic surgery for T2DM with BMI < 35 kg/m^2^ in Asia

Author/Year	Country	BMI (kg/m2)	Duration of 2TDM (years)	Procedure	No.	Age	Conclusion	Type of study
Du 2016 [23]	China	LRYGB 31.20 ± 3.4 LSG 32.1 ± 2.8	LRYGB 5.0 ± 4.2 LSG 3.5 ± 3.4	LRYGB LSG	LRYGB 64 (21:43) LSG 19 (4:15)	LRYGB 42.3 ± 9.4 LSG 39.2 ± 9.0	Both LSG and LRYGB are safe and effective bariatric procedures for T2D with diabetes and BMI <35 kg/m^2^	Cohort Study
Di 2016 [24]	China	28.2 ± 1.2	8.9 ± 5.2	LRYGB	66 (30:36)	50.4 ± 11.4	RYGB resulted in significant clinical and biochemical improvements in Chinese patients with BMI 25–30 kg/m^2^ and T2MD: 3 years	Case Series
Gong 2016 [25]	China	26.5 ± 1.4	> 7.3 ± 4.9	LRYGB	31 (14:17)	46	LRYGB is safe and effective for T2DM patients with BMI < 28 kg/m^2^	Case Series
Kular 2016 [26]	India	30–35	6.5 ± 3.1	MGB	128 (46:82)	41.6 ± 10.2	MGB provides good, long-term control of T2DM in patients with class I obesity. Early intervention results in higher remission rates.	Case Series
Li 2016 [27]	China	24–30	9.2 ± 8.1	LJISSA	57 (23:34)	43.1 ± 16.3	LJISSA seems to be a promising procedure for the control of T2DM	Case Series
Yang 2015 [28]	China	SG: 31.8 ± 3.0 LRYGB: 32.3 ± 2.4	SG:4.0 ± 1.7 LRYGB:4.2 ± 1.9:	SG LRYGB	SG:32 (9:23) LRYGB: 32 (12:19)	SG: 40.4 ± 9.4 LRYGB:41.4 ± 9.3	In this three-year study, SG had similar positive effects on diabetes and dyslipidemia compared to RYGB in Chinese T2DM patients with BMI of 28–35 kg/m^2^	RCT
Yi 2015 [29]	China	LRYGB:25.7 ± 0.9 LRYGBS:26.9 ± 0.7	LRYGB:5.9 ± 4.5 LRYGBS:6.1 ± 4.7	LRYGB LRYGBS	LRYGB:30 (22:8) LRYGBS:30 (24:8)	LRYGB:48.2 ± 8.2 LRYGBS:49.1 ± 6.2	Both procedures are effective treatments for T2DM patients with BMI < 35 kg/m^2^. LRYGB with a small gastric pouch is more suitable for Chinese diabetic patients with BMI <35 kg/m^2^.	RCT
Kim 2014 [30]	Korea	25.3 ± 3.2	9.6 ± 5.2	LSAGB	107 (53:54)	46 ± 11	After LSAGB surgery in non-obese T2DM patients, the control of T2DM was possible safely and effectively.	Case Series
Shrestha 2013 [31]	China	26.71 ± 0.69	< 10	LRYGB	33 (24:9)	49.51 ± 1.33	An improvement in postsurgical insulin sensitivity, after LRYGB even in low BMI patients with T2DM.	Case Series
Lakdawala 2013 [32]	China	30–35	8.4 (3.5–14.5)	LRYGB	52 (27:25)	49 (20–65)	LRYGB is a safe, efficacious, and cost-effective treatment for uncontrolled T2DM in patients with a BMI of 30–35 kg/m^2^	Case Series
Wu 2013 [33]	China	30.15 ± 1.73	4.9 ± 2.7	LRYGB	8 (2:6)	42.25 ± 9.95	Roux-en-Y gastric bypass has a beneficial effect on weight loss and glucose metabolism in obese type 2 diabetes patients with lower BMI	Case Series
Zhu 2012 [34]	China	26.20 ± 3.56	5.98 ± 4.54	LRYGB	30 (22:8)	48.16 ± 3.56	LRYGB is beneficial for non-obese T2DM patients in China	Case Series
Huang 2011 [35]	Taiwan, China	30.81 (25.00–34.80)	6.57 (1–20)	LRYGB	22 (2:20)	47 (28–63)	Early intervention in low-BMI patients yields better remission rates because age, BMI, and duration of T2DM predict glycemic outcomes.	Case Series
Lee 2011 [36]	Taiwan, China	30.1 ± 3.3	5.4 ± 5.1	LRYGB	62 (24:38)	43.1 ± 10.8	Laparoscopic gastric bypass facilitates immediate improvement in the glucose metabolism of inadequately controlled non-severe obese T2DM patients, and the benefit is sustained up to 2 years after surgery	Case Series
Shah 2010 [37]	India	28.9 ± 4.0 kg/m2	8.7 ± 5.3	LRYGB	15 (8:7)	45.6 ± 12	LRYGB safely and effectively eliminated T2DM in Asian Indians with BMI < 35 kg/m^2^	Case Series
Lee 2008 [38]	Taiwan, China	31.7 ± 2.7	NS	LMGB	44 (6:38)	39.0 ± 8.9	Despite a slightly lower response rate of T2DM treatment, patients with BMI < 35 kg/m^2^ still had an acceptable DM resolution, and this treatment option can be offered to this group of patients.	Case Series

*LRYGB* Laparoscopic Roux-en-Y Gastric Bypass, *VFA* Visceral fat area, *MGB* Mini-gastric bypass, *LJISSA* Laparoscopic jejunoileal side-to-side anastomosis, *LMGB* laparoscopic mini-gastric bypass, *LSAGB* Laparoscopic single anastomosis gastric bypass

**Table 3:** The major outcomes of clinical study on laparoscopic metabolic surgery for T2DM with BMI < 35 kg/m^2^ in Asia

Author/Year	Comorbidity	M	Diabetes remission rate	R/C	Major outcomes	Major Complications	Follow-up
Du 2016 [23]	Hypertension, Dyslipidemia Hyperuricemia Sleep apnea	0	LRYGB/LSG: 75%/78.9% at 1-year; 57.4% /52.9% at 3-year.	0	BMI, FPG, 2 h-PG, HbA1C, FCP, 2 h-CP, FINS, HOMA-IR	one Incomplete intestinal obstruction, one mild upper gastrointestinal bleeding	All completed 1 year follow-up, 3 were lost at 2 years, 2 lost at 3 years.
Di 2016 [24]	Hypertension, hyperlipidemia	0	74.2% (49/66) at 1-year; 57.6% (38/66) at 3-year	0	BMI, FPG, 2 hPG, HbA1C, FCP, FINS, HOMA-IR	No state	All completed 3 years follow-up
Gong 2016 [25]	Hypertriglyceridemia, Hypertension, Retinopathy Peripheral neuropathy	0	93.5% (29/31) at 6-month	0	BMI, FPG, HbA1c, CP, FINS, GLP-1	No severe complications	All completed at 1, 3 and 6 months follow-up
Kular 2016 [26]	No state	0	1, 2, 5 and 7 years were 81.8% (18/22), 78.9% (30/38),70.3% (57/81) and 68.5% (74/108)	0	BMI, waist, HbA1c, EWL, mean weight	excessive postoperative suture line bleeding with shock, anastomotic ulceration, anemia, low albumin, bile reflux, excess weight loss	Only 16%(128) of patients lost to follow0up after 7 years
Li 2016 [27]	No state	0	59.6% (34/57) at 1-year	0	FPG, 2hPBG, HbA1C, BMI, 1 h C-P	one early hemorrhage	All at 1 year follow-up
Yang 2015 [28]	Hypertension	0	SG/RYGB: 78.6% (22/32)/ 85.2% (23/32) at 3-year	0	HbA1c, FBG, CP, BMI	Two gastroesophageal reflux, a anemia	55 patient completed 3 years follow-up
Yi 2015 [29]	No state	0	LRYGB/LRYGBS: 30% (9/30)/47% (14/30)	0	BMI, HOMA-IR, HbA1c, FPG, FCP, 2hCP	10 marginal ulcers, two gastrointestinal hemorrhage	All at 1 year follow-up
Kim 2014 [30]	No state	0	53%, 63%, 90% at 1, 2 and 3 year	1^a^	BMI, HbA1c, Fasting glucose, 2 h glucose, CP, Insulin, HOMA-IR	Postoperative bleeding, outflow stasis, infected fluid collection, leakage	144 at 1 year; 116 at 2 year, 51 at 3 year
Shrestha 2013 [31]	No state	0	A significant decrease in the levels of FPG, 2 h PG, HbA1c, and TG	0	BMI, HbA1c, FPG, 2hPG, FINS, HOMA-IR	No state	All at 3 month follow-up
Lakdawala 2013 [32]	Hypertension, dyslipidemia, hyperuricemia, gastroesophageal reflux disease, sleep apnea, joint pain	0	96.2% (50/52) at 1 year and 5 year	0	Average blood glucose, FINS, postprandial serum insulin, FCP, HbA1c	one seromas and one nausea, no major complication	4d, 1,3,6, months, 1, 2, 3, 4, 5 years
Wu 2013 [33]	No state	0	75% at 2 months, 83.3% at 4 months	0	BMI, FPG, 2hPG, HbA1c	two vomiting and diarrhea, two gastric fistula and infection	All at 2 months, 6 at 4 months.
Zhu 2012 [34]	Chronic gastritis, fatty liver, hypertension, hypertriglyceridemia, diabetic retinopathy, diabetic nephropathy	0	Significant reduction in Glycosylated hemoglobin, diabetes was completely resolved in 9 cases	0	BMI, Waist-hip ratio, FPG, 30 m PG, 2 h PG, HbA1C	No major complication	All at 12 months follow-up
Huang 2011 [35]	Hyperlipidemia, hypertension, steatohepatitis, gouty arthritis	0	90.9% (20/22) at 12 months.	0	BMI, Glucose, HbA1, ctriglyceride	No state	All at 12 months follow-up
Lee 2011 [36]	No state	0	0%,11%, 37%, 53%, 57%, and 55% patients in 1, 4, 12, 26, and 52 weeks and 2 years	0	BMI, Glucose, insulin, HbA1c, HOMA, Glucose, Insulin, Insulinogenic index	seven minor complications, no major complication	62 at 1,4 week, 45 at 12 week, 40 at 26 weeks, 30 at 52 weeks, 20 at 2 years.
Shah 2010 [37]	Hypertension, dyslipidemia	0	80% (12/15)at 3 months	0	BMI, Waist circumference, Fasting blood glucose, HbA1c	No major complication	All at 6, 9 months
Lee 2008 [38]	2 comorbidity	0	76.5% (34/44) at 1 year	0	BMI, glucose, T-chole, Triglyceride, insulin, CP, HbA1c	No major complication	At 1 year

*M* mortality, *R/C* Reoperation/conversion to open, *BMI* Body mass index, *FPG* fasting plasma glucose, *PG* plasma glucose, *HbA1C* glycated hemoglobin, *FCP* fasting C peptide, *CP* c-peptide, *FINS* Fasting insulin, *HOMA-IR* homeostatic model of assessment-insulin resistance, *GLR-1* glucagon-like peptide-1, *EWL* excess weight loss, *PBG* postprandial blood glucose

^a^conversion to open surgery

## Discussion

This scoping review identified 205 studies of laparoscopic metabolic surgery for T2DM in Asia and performed an evidence-based analysis. Most of the studies were clinical research (78.0%) and published in English (94.1%) with increasing numbers of publication each year during the past decade. Using the *Oxford Center for EBM Levels of Evidence*, only 12.1% of the studies were of Level I evidence, mostly conducted in East Asia (62.1%). LSG was the most commonly reported surgical procedure, accounting for 64.9% in Asian countries. All studies reported that laparoscopic metabolic surgery was safe and effective for T2DM with or without obesity. There were 16 studies on T2DM with BMI of > 25 kg/m^2^ to < 35 kg/m^2^, mostly from China. 70.2% procedures were LRYGB [23–38]. Our review suggests that laparoscopic metabolic surgery might be a safe and effective treatment in T2DM patients with BMI from 25 kg/m^2^ to 35 kg/m^2^.

This study suggests that the use of laparoscopic metabolic surgery for T2DM is increasing in Asia, especially in China and India. This may be due to the increasing number of obese patients in this region. Adela Hruby et al. [39] reported that from 1980 to 2010, in most Asian countries and region, the incidence of obesity and overweight has increased in both children and adults. Obesity is highly prevalent in China and India, representing a high proportion of the total number of people with obesity and diabetes globally [40]. The role of laparoscopic metabolic surgery for obesity with T2DM has been included in many national guidelines in Asia and other regions globally [41–43]. The recent increases in funding investments in medical research in China could also be a contributing factor to the increase in the number of studies. The total number of international publication in China has ranked second in the world in 2015, which may indicate that there is more scientific activities being conducted in China [44]. It is reported that there were 50 million bariatric surgeries globally in 2014, LSG accounting for 51.7%, RYGB 26.8%, BPD-DS 11.5%, and AGB 9.5% [45]. The 3rd annual conference of Chinese Society for Metabolic and Bariatric Surgery in 2015 reported that in China, LSG accounted for 65.5% and LRYGB 28.9% of bariatric procedures.

Previous studies have shown that LSG is a low-risk, easy to perform, faster, less expensive, and more effective weight loss procedure with lower complication rates, and may have a better safety profile in the short-term compared to LRYGB [46–48]. However, Li JF et al. [49] suggested that LRYGB is more effective than LSG for the treatment of T2DM and metabolic syndrome. Yang et al. [27] reported in a RCT of 64 patients that both LSG with LRYGB procedures achieved complete remission of T2DM with glycated hemoglobin (HbA1c) <6.0% without taking any diabetic medications. The LRYGB group had significantly greater weight loss than the SG group (*p* = 0.017). A cohort study among 304 patients indicated that LRYGB showed improved effectiveness in T2DM resolution when compared to LSG at a 5-year follow-up assessment [50]. Evidence also suggests that LRYGB could rapidly improve insulin resistance and associated pancreatic beta-cell function [51, 52]. And both LSG and LRYGB have been suggested as safe and effective bariatric procedures for obesity [53]. Therefore, laparoscopic metabolic surgery has been widely recommended as the most effective treatment for T2DM patients with BMI ≥ 35 kg/m^2^ or in patients with one or more severe obesity-related co-morbidities (T2DM, hypertension, hyperlipidemia, obstructive sleep apnea, etc.) [54–57]. Laparoscopic metabolic surgery also be considered to be an option to treat T2MD in patients with BMI 30.0–34.9 kg/m^2^ or 27.5–32.4 kg/m^2^ in Asian descent, and inadequately controlled hyperglycemia despite optimal medical treatment by either oral or injectable insulin during Diabetes Surgery Summit II [54].

This investigation reveals interesting results that the research of laparoscopic metabolic surgery for T2DM with BMI of > 25 kg/m^2^ to < 35 kg/m^2^ has increased in Asian countries (Fig. 2; Table 3), and all studies had an acceptable diabetes mellitus resolution (remission rate range from 52.9 to 96.2% at 1, 3, or 5 years). A meta-analysis including nine trials (from China, USA, Chile, Brazil and Spain) with T2DM and BMI < 35 kg/m^2^ undergoing LRYGB, suggested that these patients had a significant decrease in BMI (*P* < 0.00001), remission of diabetes (glucose, *P* < 0.00001, hemoglobin A1c, *P* < 0.00001) at 12 months follow-up [58]. Lee et al. [38] investigated 201 patients with impaired fasting glucose or T2DM undergoing laparoscopic mini-gastric bypass, and showed that 76.5% patients with 25 kg/m^2^ < BMI < 35 kg/m^2^ met treatment goals (HbA1C < 7%, LDL < 150 mg/dl, triglyceride <150 mg/dl) compared with 92.4% patients with BMI > 35 kg/m^2^. Although, all 16 studies in our review showed that laparoscopic metabolic surgery is effective for patients with BMI of > 25 kg/m^2^ to < 35 kg/m^2^ in Asian countries, further research is needed to determine whether these effects are universal across other countries. Moreover, there were no mortalities nor severe complications reported in any of trials (Table 3) [23–38]. And the Table 3 showed that most surgery for T2DM with low BMI were performed in East Asia, which is correlated with a lower BMI than in European/western patients, the risk of T2DM in different ethnics is different. In Asia, the related complication of T2DM occurred when BMI is not high. This might also influences indication for “diabetes-surgery” [18, 59–61].

This review has some limitations: Firstly, this study only analyzed the Asian literature from a single database, PubMed, and might not include studies published in other regional or national databases. Secondly, we could not perform a meta-analysis in our present study to compare the differences between low and high (BMI > 25 kg/m^2^ to < 35 kg/m^2^ vs BMI ≥ 35 kg/m^2^) and other characteristics due to heterogeneity of the reports. Thirdly, there is a lack of well-designed rigorous randomized controlled trials investigating this intervention, and therefore a paucity of high-level evidence to support any recommendations. Therefore, further research is obligatory to understand long-term health outcomes, improvements and sustainability of the glycemic control and advance surgical procedures.

## Conclusions

This review demonstrated that SLG and LRYGBP are the two commonly used surgical procedures for T2DM patients with comorbid obesity in Asia. Laparoscopic metabolic surgery can be performed in T2DM patients with BMI of > 25 kg/m^2^ to < 35 kg/m^2^. However, there is a need for further research to identify long-term complications, sustained rates of diabetes resolution, and promotion of the further development of metabolic surgery in Asia.

## Abbreviations

BMI: Body Mass Index
BPD-DS: Laparoscopic biliopancreatic diversion with duodenal switch
EBM: Evidence-based medicine
IDF: International Diabetes Federation
LAGB: Laparoscopic adjustable gastric banding surgery
LRYGB: Laparoscopic Roux-en-Y gastric bypass
LSG: Laparoscopic sleeve gastrectomy
RCT: Randomized control trial
T2DM: Type 2 diabetes
